# Supplementary figures and images for: Wild-type bone marrow cells repopulate tissue resident macrophages and reverse the impacts of homozygous CSF1R mutation
Source: PLoS Genet. 2025 Jan 27;21(1):e1011525. doi: 10.1371/journal.pgen.1011525 (PMC11785368; doi:10.1371/journal.pgen.1011525)

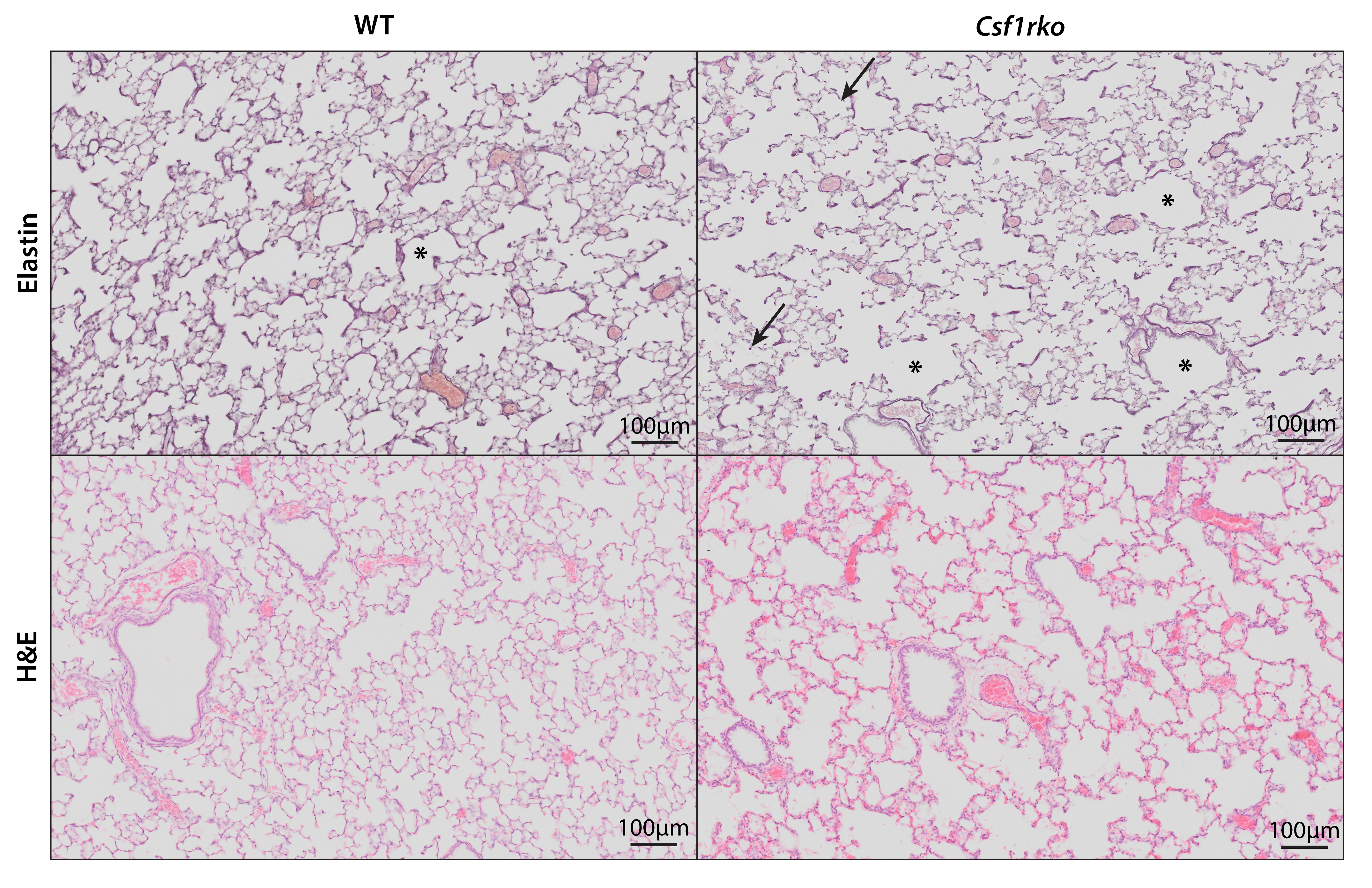

Supplement: S1 Fig — Representative H&E and elastin images of 3-week-old WT and Csf1rko lung. Lungs were expanded and flushed with PBS via the trachea prior to fixation. * indicates examples of terminal bronchioles. Arrows point to regions of alveolar wall destruction, as defined by a board certified veterinary anatomic pathologist. Original magnification: 40X. Scale bar: 100μm. (TIF) [file pgen.1011525.s001.tif]

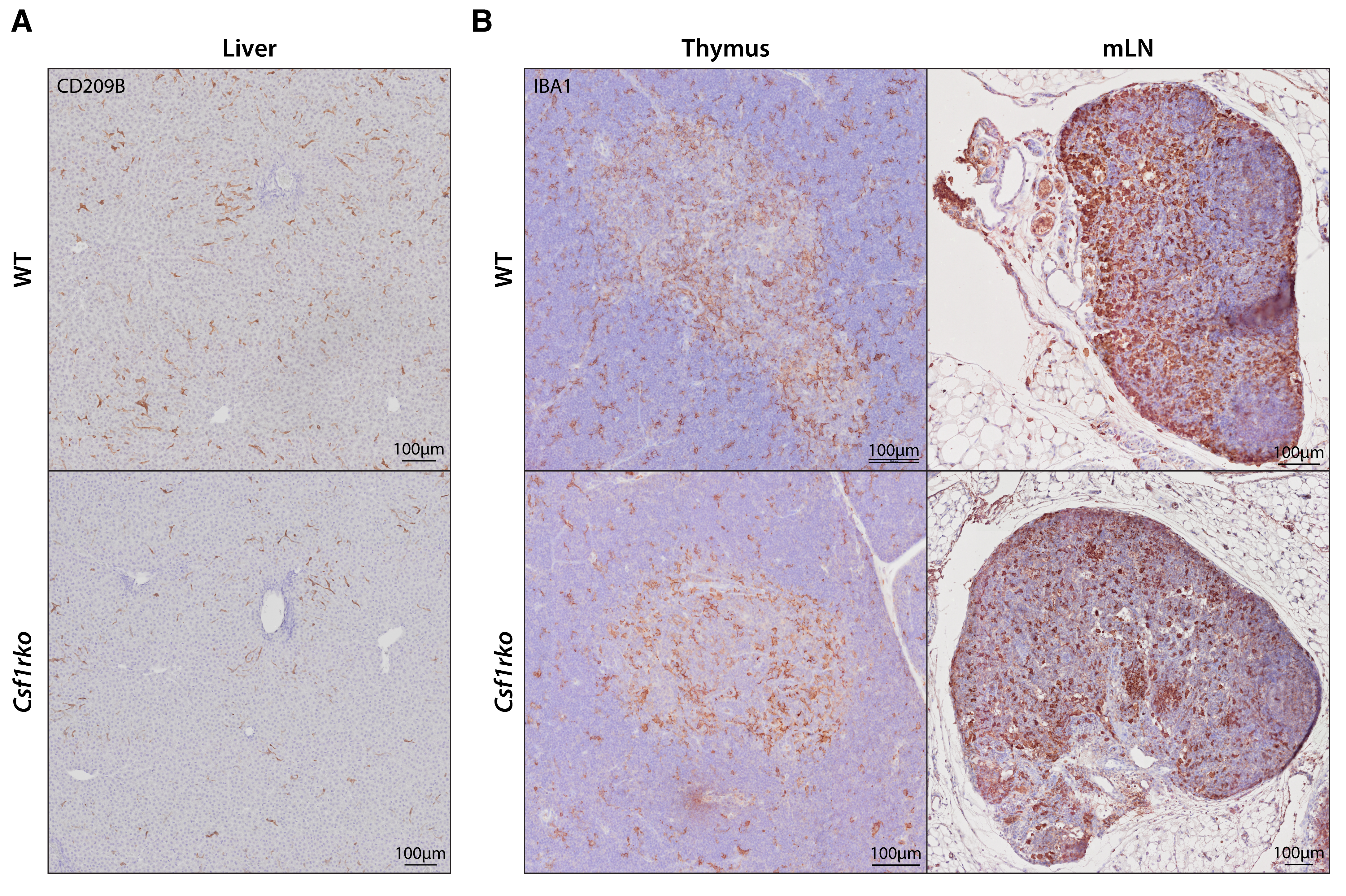

Supplement: S2 Fig — (A) Representative images showing immunohistochemical localization of CD209B (brown) in 3 week WT and Csf1rko liver. Scale bar: 100μm. (B) Representative images showing immunohistochemical localization of IBA1 (brown) in 3 week WT and Csf1rko thymus and mesenteric lymph node (mLN). Scale bar: 100μm. (TIF) [file pgen.1011525.s002.tif]

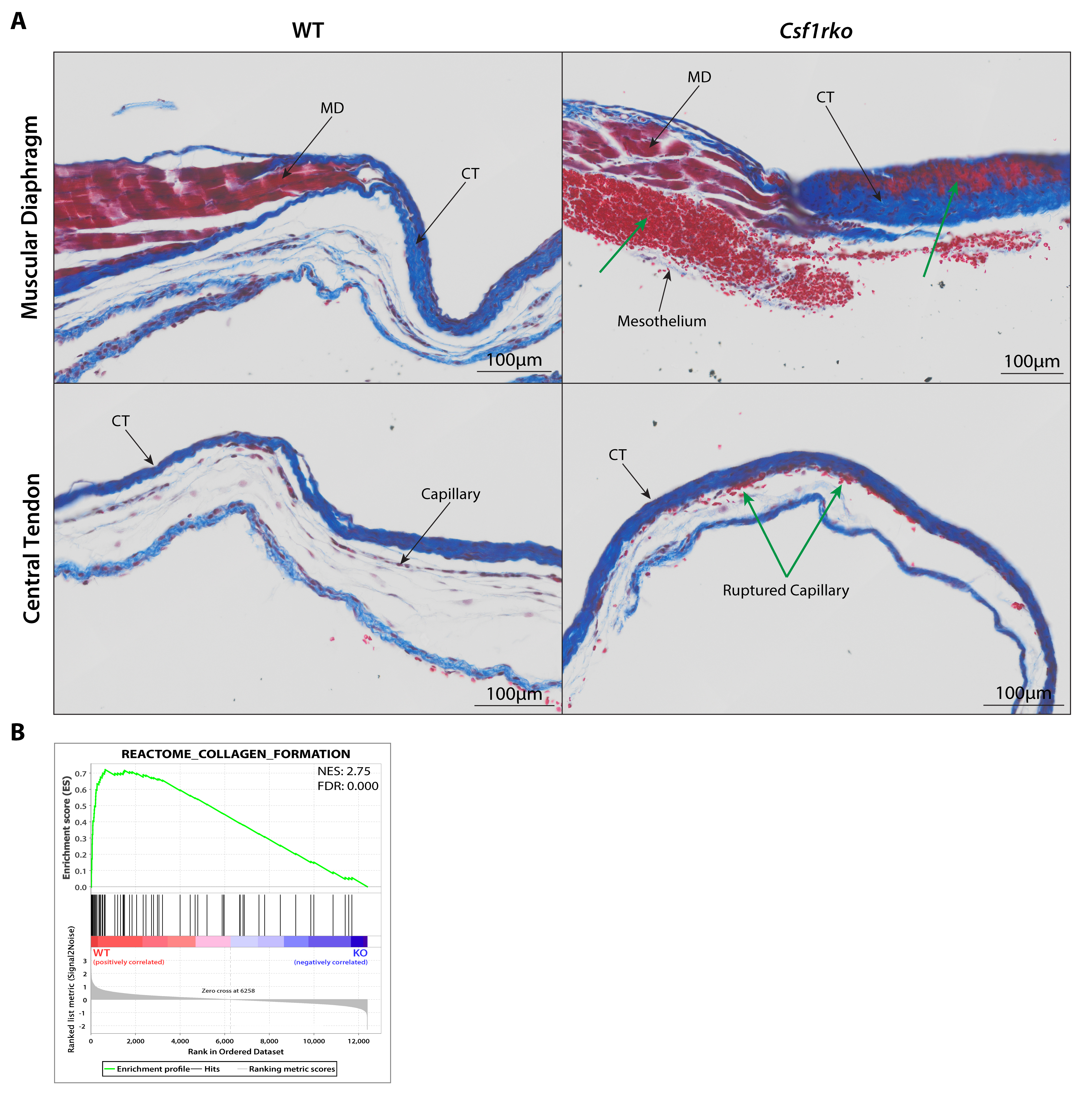

Supplement: S3 Fig — (A) Representative Masson’s Trichrome images of 3 week WT and Csf1rko diaphragms. MD: muscular diaphragm, CT: central tendon. Muscular diaphragm region is showing the junction between muscular diaphragm and central tendon. Green arrows indicate regions of haemorrhage in both muscular diaphragm and the dense/loose connective tissue of the central tendon of Csf1rko. Scale bar: 100μm. (B) Gene set enrichment plot showing a reduction in collagen related genes in the Csf1rko diaphragm. Created using the C2 (curated gene sets) collection from the Molecular Signatures Database (MSigDB). NES – Normalised Enrichment Score; FDR – False Discovery Rate; positive enrichment score indicates an enrichment in the WT animals and a negative enrichment in the Csf1rko animals. (TIF) [file pgen.1011525.s003.tif]

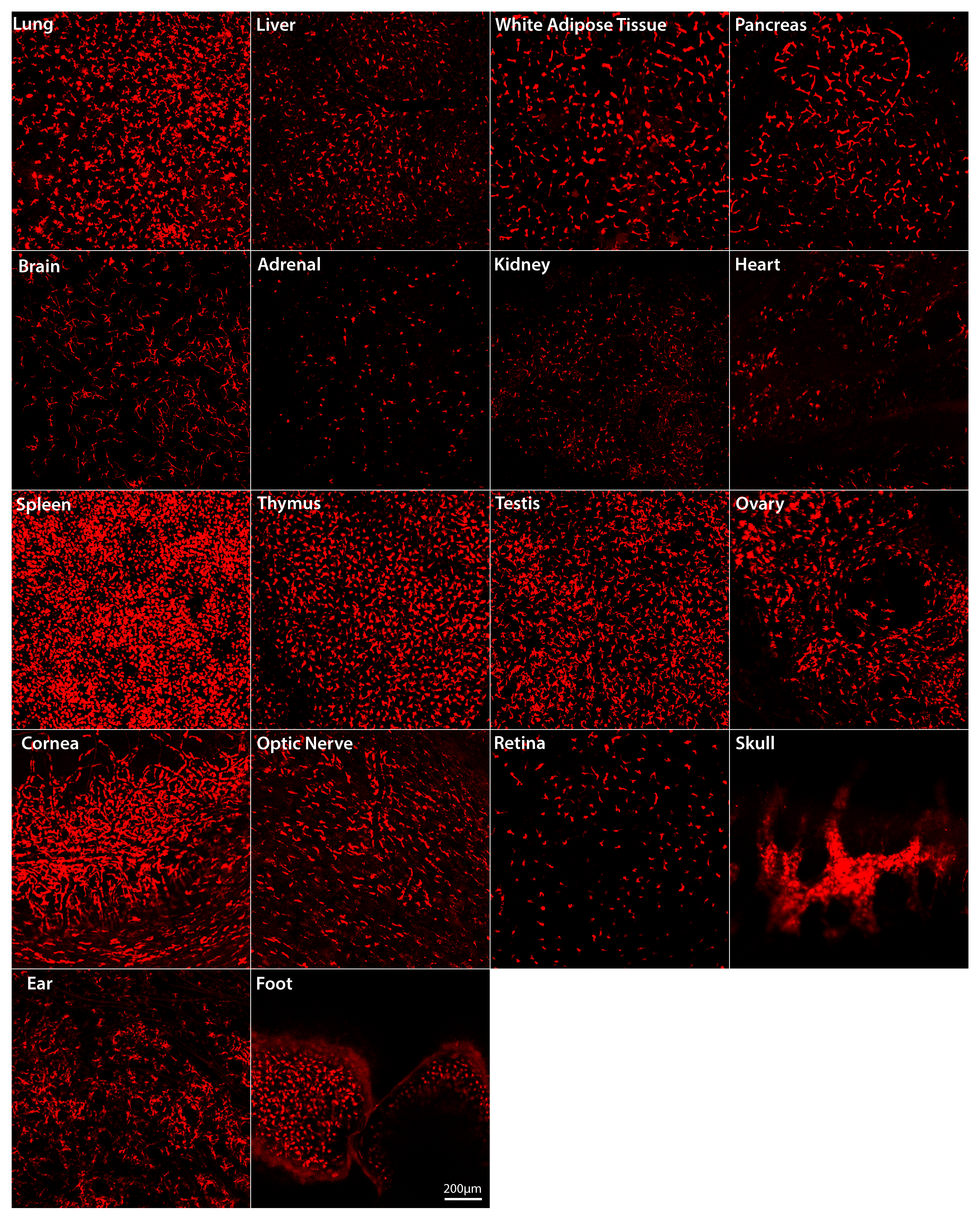

Supplement: S4 Fig — Representative whole-mount immunofluorescence images of tissues from Csf1rko rats 12 weeks after IP transfer of WT Csf1r-mApple donor bone marrow cells. No fluorescent signal is detected in any tissue in nontransgenic WT rats or in untransplanted Csf1rko rats. Scale bar: 200μm. (TIF) [file pgen.1011525.s004.tif]

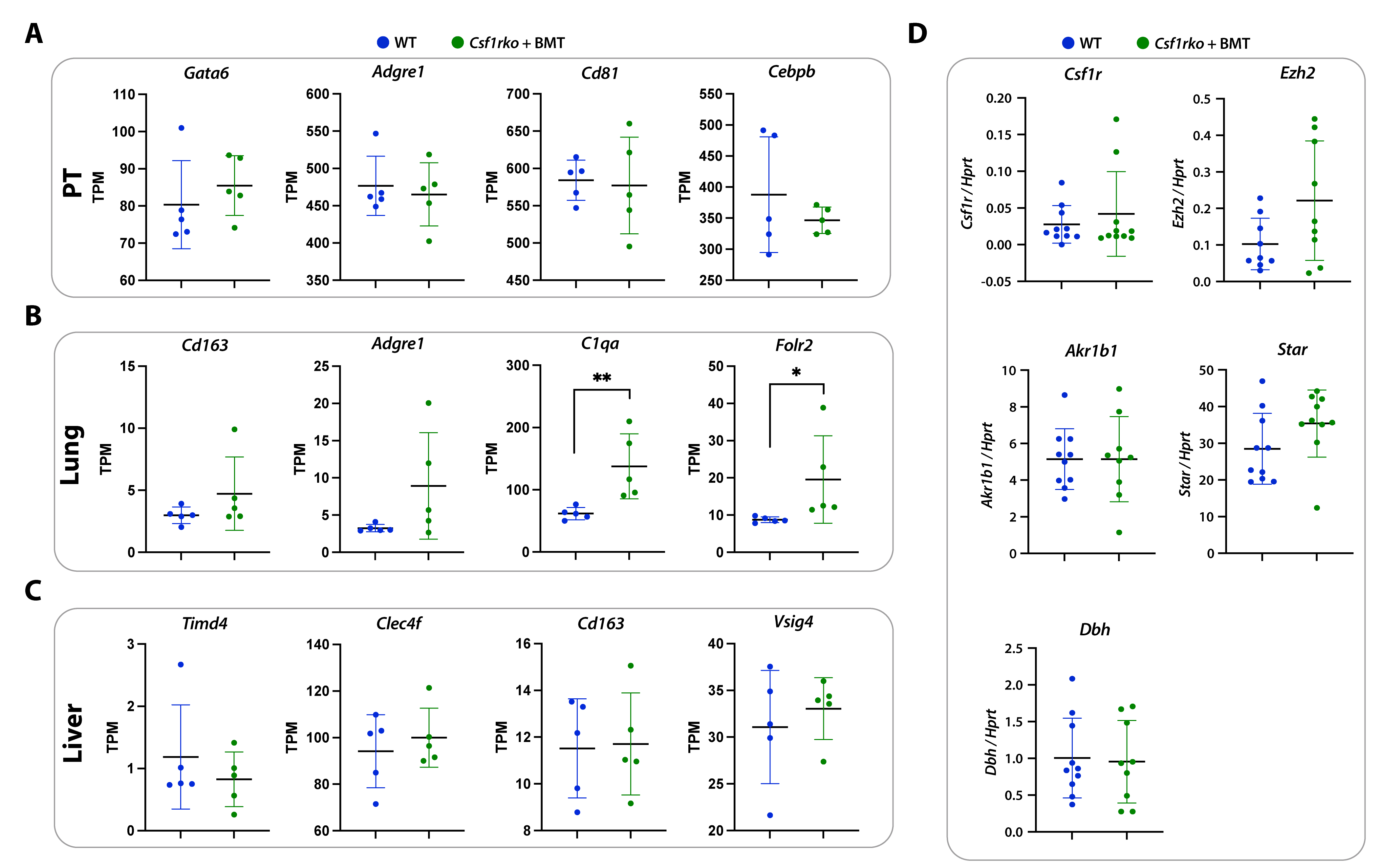

Supplement: S5 Fig — (A–C) Expression profiles in transcripts per million (TPM) for selected genes from RNA–Seq data of (A) peritoneal lavage (PT)(B) lung and (C) liver from WT and Csf1rko BMT recipients. Primary data is in S9 Table. Graphs show the mean ± SD. (D) qRT–PCR analysis of adrenal glands from WT and Csf1rko BMT recipients (10 WT; 9-10 Csf1rko + BMT). Graphs show the mean ± SD. *, P < 0.05; **, P < 0.01; ***, P < 0.001; ****, P < 0.0001. (TIF) [file pgen.1011525.s005.tif]

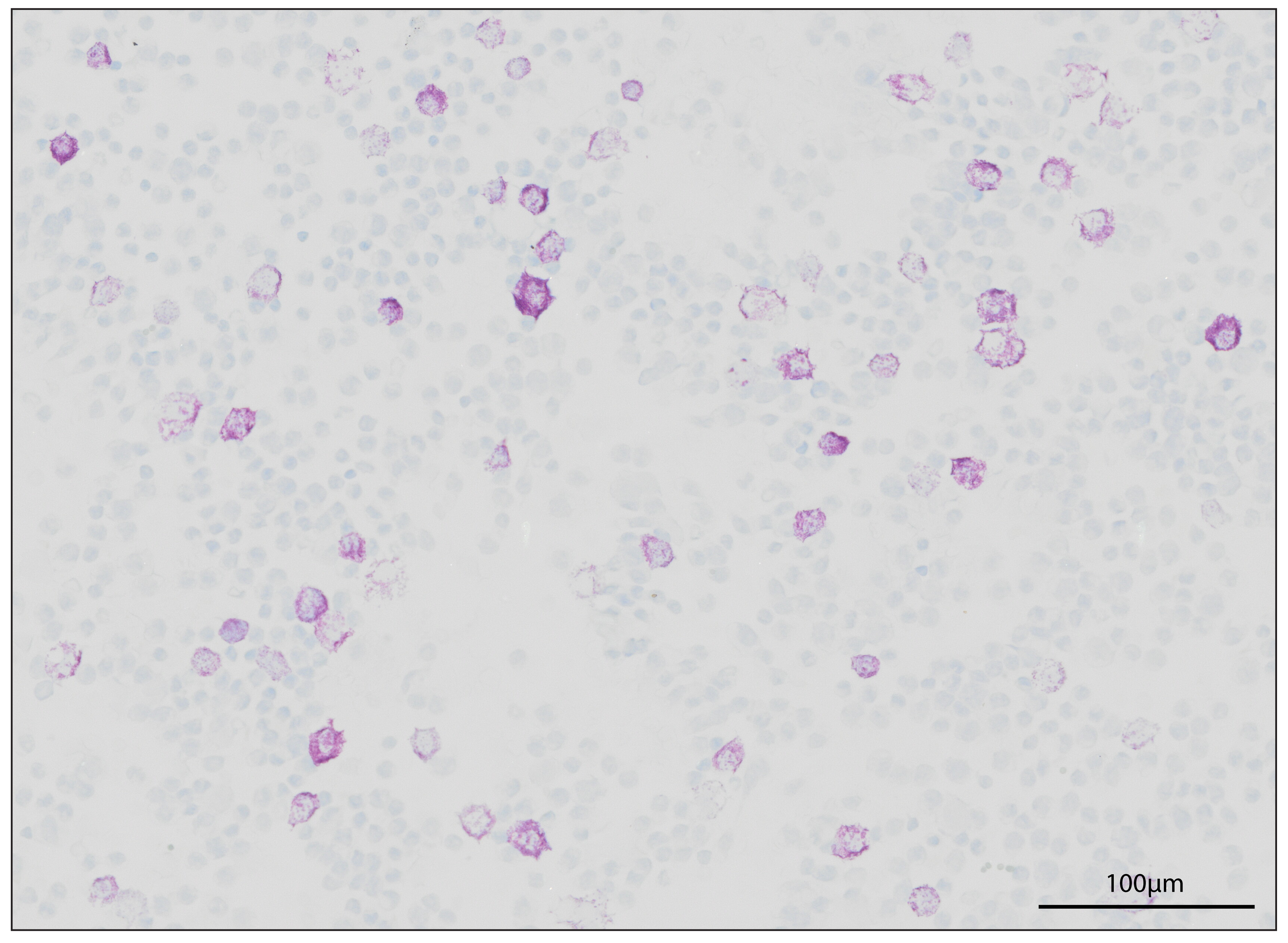

Supplement: S6 Fig — Scale bar: 100μm. (TIF) [file pgen.1011525.s006.tif]

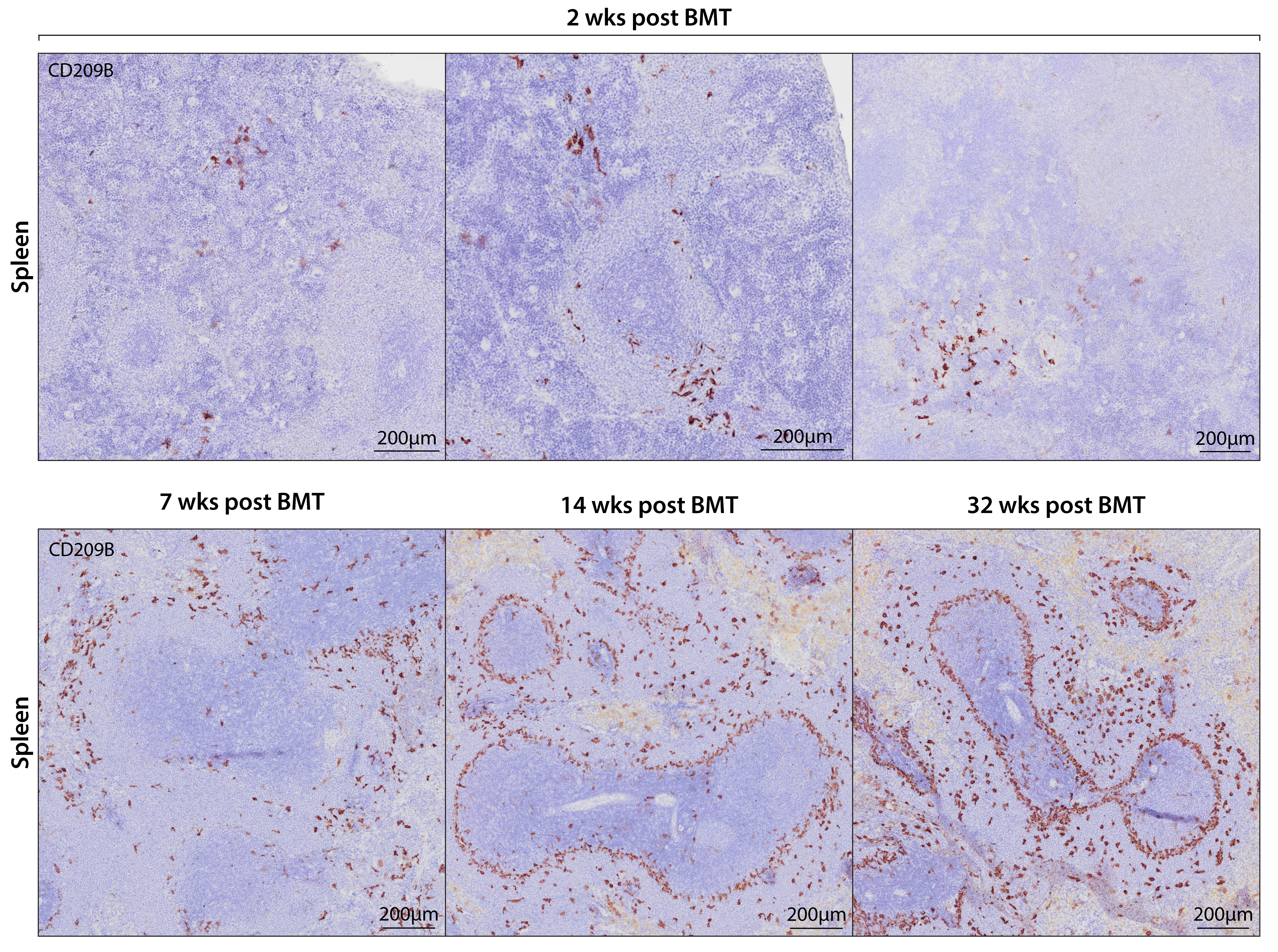

Supplement: S7 Fig — Representative images showing immunohistochemical localization of CD209B (brown) in Csf1rko BMT recipient spleens at different time points post BMT. 3 different regions of the same spleen are shown for 2 wks post BMT. Scale bar: 200μm. Original magnification for all images: 40X. (TIF) [file pgen.1011525.s007.tif]
