# Supplementary material for: Wild-type bone marrow cells repopulate tissue resident macrophages and reverse the impacts of homozygous CSF1R mutation
Source: PLoS Genet. 2025 Jan 27;21(1):e1011525. doi: 10.1371/journal.pgen.1011525 (PMC11785368; doi:10.1371/journal.pgen.1011525)
Supplement: Attachment — Submitted filename: Response to reviewers.docx [file pgen.1011525.s018.docx]

**Note:** Line numbers mentioned here refer to the “Revised Manuscript with Track Changes” document

**Reviewer 1.**

Carter-Cusack et al. provide a comprehensive, descriptive study on Csf1rko rats, both reconstituted with and without BM. It is encouraging to see that the macrophage population in rats resembles the gene transcript profiles of mice and humans. Among their findings, C1q emerged as a prominent gene signature, strongly associated with interstitial macrophages. Notably, interstitial macrophages do not develop in the absence of CSF1, as demonstrated in studies by Ural et al. (Sci Immunology, 2020) on the lung and Lim et al. (Immunity, 2018; 10.1016/j.immuni.2018.06.008) on the heart. Thus, the loss of gene signatures outlined in this study unmistakably points to interstitial macrophages, a population present across various organs and species. Recommended additional papers for assessing interstitial macrophage genes across various organs and species include the following:

Plantinga M, et al., Immunity 2013
Tamoutounour S et al., Immunity 2013
Epelman S, et al., Immunity 2014
Gibbings et al., AJRCMB 2017
Chakarov, et al., Science 2019
BB Ural et al., SciImmu 2020
Schyns, et al., NatCom 2019
Dick et al., SciImmu 2022
Xi Li, et al, Nature Imm 2024

We are aware of this literature. The senior author first described interstitial macrophages using the F4/80 marker in the 1980s and recently published a meta-analysis of published mouse RNA-seq data. We had not considered cross-species comparison in detail. We have added additional text to the discussion extending the background and highlighting some of differences in macrophage transcriptome profiles between the rat and mouse.

**Reviewer 2.**

We thank the reviewer for the constructive suggestions to improve the manuscript.

While the analysis demonstrates CSF1R dependency across tissues, particularly non-lymphoid organs, Figure 1 could benefit from additional annotations that highlight critical findings. For instance, identifying clusters with substantial CSF1R-dependent gene expression changes would strengthen the figure's biological relevance, especially regarding key clusters such as the macrophage-associated genes found in subsequent analyses.

Figure 1 has been expanded to provide more context and highlight additional clusters of interest. Figure 1B now includes labels to clusters other than the Csf1r-dependent macrophage cluster. Figure 1C has been added to show the expression profiles of these clusters.

The immunohistochemistry images in part (E) of Figure 3 are labeled, but it could be more informative if the text explicitly explained the cellular localization or morphological distinctions shown between WT and KO groups. The authors should put arrows on the Figure 3E to indicate the WT and KO differences.

Figure 3E has been amended to include additional annotations to aid in the explanation of the morphology and cellular localisation. The text has been changed to make it clearer that the CD163^+^ cells are completely lost in the *Csf1rko* animals (lines 218-220).

Figure 4 demonstrates the immunohistochemical localization of CD209B in the thymus, spleen, and mesenteric lymph nodes at 40X magnification with a 100µm scale bar, yet it lacks clarity on notable qualitative or quantitative differences across tissues, which would help distinguish each representation . Additionally, the volcano plot showing differential expression (DE) between WT and Csf1rko lymph nodes, calculated using DESeq2 with log fold changes adjusted via lfcShrink, could benefit from more detailed context on specific genes or clusters impacted by this DE analysis to enhance understanding of key findings.

Figure 4 has been changed to include zoomed in regions of each tissue to provide a clearer view of the cellular localisation of the CD209b^+^ macrophages. Additional annotations have been added to these images to provide context to the tissue structures. Quantification was not performed on this staining. The modified images show CD209b^+^ macrophages are entirely absent in the *Csf1rko*. We have added a sentence in results pointing to the more detailed of the function of Csf1r-dependent macrophages in antigen sampling in the discussion

Figure 5 discusses gene expression profiles in WT and Csf1rko samples across gut tissues (colon, ileum, and duodenum) but lacks clarity on gene selection criteria and specific affected clusters, which would aid comprehension. It groups Csf1r-dependent genes into those common across all gut tissues, those more impacted in the colon and duodenum, and DC-associated genes elevated in the Csf1rko ileum, though further explanation of this segmentation’s relevance to the study is needed. Additionally, Figure 5D’s immunohistochemical staining for peripherin in the ileum and its quantification require more context to clarify its role in neuronal development or gut function within the Csf1rko model.

An addition panel has been added to Figure 5 (Figure 5A) to clarify the reasoning for selecting the specific genes shown in the following panels. Figure 5A shows the Csf1r dependent cluster in all 3 regions of gastrointestinal tract. The genes in this cluster follow slightly different patterns of expression in each region, and this is shown in panels 5B and 5C. A subset of the genes was undetectable in all regions of gut in the *Csf1rko* (Figure 5B), while another subset was maintained to a certain degree in the ileum (Figure 5C). The reference to classical DC markers (Figure 5D) has been referenced and clarified in the revised text.

Additional annotations have been added to the peripherin staining in Figure 5E to provide a clearer view of the structures within the tissue. Text has also been added to provide more context about the proposed roles of macrophages in the gut and about what peripherin is and why it is being used as a marker (Lines 323-334).

In Figure 6, the authors fails to provide sufficient context or clarity regarding specific findings. Details about the methods used and the significance of the results, such as whether the observations align with prior data, are minimal. Additionally, explanations connecting visual elements (like particular markers or staining patterns) to study conclusions are sparse, making it difficult for readers to understand the implications. Including these contextual details would enhance the text’s clarity and strengthen the link between visual data and the conclusions drawn.

Substantial changes have been made to Figure 6 to provide more clarity, and to more strongly link the results text to the figure panels. Cluster expression plots and gene ontology term annotation are now shown in Figure 6A for clusters 1, 4, and 5. This is to strengthen the statements made in the text that these clusters are involved in protein synthesis, satellite cell expression, and mature muscle function. When taken together, these new figures provide more context to the conclusion that the *Csf1rko* skeletal muscle is developmentally delayed compared to wild type. The full tables of gene ontology analysis for clusters 1, 4 and 5 have been added to Table S5 (Table S5B-D).

More background information has been given in the text regarding the reason for looking at Schwann cells and peripheral myelination in these animals (Lines 362-374).

Additional annotation has been added to Figure 6D to better show the structures in question, and the text has been supplemented to provide additional analysis regarding the cause of the diaphragmatic haemorrhage (Lines 383-388).

For Figure 7, the main issues in the manuscript include a lack of clarity in the rationale for focusing on specific gene expression profiles in the kidney and adrenal gland. Additionally, there is insufficient contextual information about the functional relevance of selected genes, particularly regarding their potential impact on the organ development being studied. The calcium concentration measurement results would benefit from more detailed interpretation to strengthen the findings. The imaging details, especially for the adrenal gland, could also be improved to provide a more comprehensive understanding of the histological differences between WT and Csf1rko samples. This would enhance reader comprehension of both the structural and molecular alterations under investigation.

Figure 7 has been changed to improve the clarity in relation to the text. Network analysis cluster plots were added in Figure 7A to aid in the description of the changes to the regions of kidney associated with the *Csf1rko* (Lines 390-399). Quantification of glomeruli within the kidney in *Csf1rko* and wild type has also been added to confirm the inferred increase based on glomerular markers in the RNA-seq data.

Additional background has been given in the text regarding calcium dyshomeostasis and its relevance to the Csf1rko model (namely that these animals undergo severe osteopetrosis due to a lack of osteoclasts, and therefore lose the primary method of calcium homeostasis in the body) (Lines 400-412). This additional information provides greater context for the interpretation of the serum calcium concentration, the evidence for up-regulation of PTH responsive genes as well as the reasons for choosing the genes shown in Figure 7D.

Significant annotations have been added to the histology images of the adrenal gland in Figure 7E. This was done to clearly show the different regions of the adrenal cortex and medulla, thus highlighting how these regions differ between WT and *Csf1rko*. A substantial portion of text has been added to describe these histological differences more clearly (Lines 418-424).

The main concerns regarding the descriptions for Figure 8 focus on the clarity and depth of the gene expression analysis across multiple tissues. There is a need to specify better the rationale behind selecting particular genes for expression profiling in WT and Csf1rko samples and to discuss the impact of differentially expressed genes within the intestinal regions examined. Additionally, more explicit detail on tissue-specific gene clusters and their significance would enhance the figure’s interpretive value.

The reasoning behind selecting certain genes to use as examples has been clarified. For the peritoneal cavity (Lines 515-517) and liver (Line 534), references have been added to provide reasoning for the selection of certain genes. For the lung, the analysis of the 3 week old WT and Csf1rko lungs showed that genes chosen were among the most correlated with the loss of Csf1r, and were therefore good markers of the interstitial macrophages that are lost (Lines 529-530).

**Reviewer 3.**

It would be helpful to discuss why circulating C1Q is undetectable in Csf1rko rats. While I understand the study is comprehensive, I recommend condensing the discussion section to focus on the main findings.

We have inserted comment noting that the loss of circulating C1q is consistent with the loss of C1qa/b/c mRNA in every tissue (Lines 483-484).

While we recognise the discussion is relatively long, we feel that the comprehensive approach we have taken raises multiple distinct issues that warrant discussion. To point more clearly to the main findings we have inserted new subheadings and modified existing subheadings in the form of definitive statements.
